# Aortic Arch Calcification and Cardiomegaly Are Associated with Overall and Cardiovascular Mortality in Hemodialysis Patients

**DOI:** 10.3390/jpm11070657

**Published:** 2021-07-13

**Authors:** Shih-Hsiang Ou, Yi-Hsueh Liu, Tung-Ling Chung, Jiun-Chi Huang, Pei-Yu Wu, Ho-Ming Su, Szu-Chia Chen

**Affiliations:** 1 Graduate Institute of Clinical Medicine, College of Medicine, Kaohsiung Medical University, Kaohsiung 807, Taiwan; blueyeou1104@gmail.com (S.-H.O.); Liuboy17@gmail.com (Y.-H.L.); karajan77@gmail.com (J.-C.H.); 2 Division of Nephrology, Department of Internal Medicine, Kaohsiung Veterans General Hospital, Kaohsiung 813, Taiwan; kaoru02323@gmail.com; 3 Department of Internal Medicine, Kaohsiung Municipal Siaogang Hospital, Kaohsiung Medical University, Kaohsiung 812, Taiwan; wpuw17@gmail.com (P.-Y.W.); cobeshm@seeed.net.tw (H.-M.S.); 4 Division of Cardiology, Department of Internal Medicine, Kaohsiung Medical University Hospital, Kaohsiung Medical University, Kaohsiung 807, Taiwan; 5 Graduate Institute of Medicine, College of Medicine, Kaohsiung Medical University, Kaohsiung 807, Taiwan; 6 Division of Nephrology, Department of Internal Medicine, Kaohsiung Medical University Hospital, Kaohsiung Medical University, Kaohsiung 807, Taiwan; 7 Faculty of Medicine, College of Medicine, Kaohsiung Medical University, Kaohsiung 807, Taiwan

**Keywords:** hemodialysis, aortic arch calcification, cardiothoracic ratio, overall mortality, cardiovascular mortality

## Abstract

Patients with end-stage renal disease have a higher risk of cardiovascular morbidity and mortality. In this study, we investigated the predictive ability of a combination of cardiothoracic ratio (CTR) and aortic arch calcification (AoAC) for overall and cardiovascular mortality in patients receiving hemodialysis. We also evaluated the predictive power of AoAC and CTR for clinical outcomes. A total of 365 maintenance hemodialysis patients were included, and AoAC and CTR were measured using chest radiography at enrollment. We stratified the patients into four groups according to a median AoAC score of three and CTR of 50%. Multivariable Cox proportional hazards analysis was used to identify the risk factors of mortality. The predictive performance of the model for clinical outcomes was assessed using the χ2 test. Multivariable analysis showed that, compared to the AoAC < 3 and CTR < 50% group, the AoAC ≥ 3 and CTR < 50% group (hazard ratio [HR], 4.576; *p* < 0.001), and AoAC ≥ 3 and CTR ≥ 50% group (HR, 5.912; *p* < 0.001) were significantly associated with increased overall mortality. In addition, the AoAC < 3 and CTR ≥ 50% (HR, 3.806; *p* = 0.017), AoAC ≥ 3 and CTR < 50% (HR, 4.993; *p* = 0.002), and AoAC ≥ 3 and CTR ≥ 50% (HR, 8.614; *p* < 0.001) groups were significantly associated with increased cardiovascular mortality. Furthermore, adding AoAC and CTR to the basic model improved the predictive ability for overall and cardiovascular mortality. The patients who had a high AoAC score and cardiomegaly had the highest overall and cardiovascular mortality among the four groups. Furthermore, adding AoAC and CTR improved the predictive ability for overall and cardiovascular mortality in the hemodialysis patients.

## 1. Introduction

End-stage renal disease (ESRD) is a major global health care issue which is associated with an increased risk of cardiovascular morbidity and mortality [1,2,3]. Abnormalities in cardiac structure and function are also known to increase the risk of cardiovascular disease [4]. The risk factors for cardiovascular disease in patients with ESRD include both traditional and non-traditional risk factors, such as diabetes mellitus, hypertension, dyslipidemia, advanced age, oxidative stress, inflammation, fluid overload, endothelial dysfunction and vascular calcification [5,6,7]. Therefore, the early identification of these risk factors in these patients is crucial.

Chest radiography is an inexpensive non-invasive method that can be used to evaluate aortic arch calcification (AoAC) and cardiothoracic ratio (CTR). AoAC is mainly used to represent the severity of vascular calcification (VC) [8]. VC is a morphological marker of vascular pathologic changes, and it is also an important predictor of cardiovascular burden [9]. An increasing number of studies have reported associations between AoAC measured on chest radiography and intima-media thickness, pulse pressure and cardiovascular events in the general population [10,11]. In patients with chronic kidney disease (CKD) or ESRD, several recent studies have also reported associations between AoAC as measured on chest radiography and diastolic left ventricular (LV) dysfunction, increased afterload, arterial stiffness, and even a higher rate of cardiovascular mortality [12,13,14]. The incidence of VC is higher in patients with ESRD than in the general population, and its impact on morbidity and mortality is greater in patients with ESRD [15]. CTR is related to LV size, and it is also easily obtained using chest radiography. A high CTR often indicates cardiomegaly, which is associated with elevated LV mass, LV hypertrophy, and even heart failure [16,17]. Although CTR lacks specificity in identifying specific structural cardiac lesions, its presence still has a strong prognostic significance in high-risk populations. In patients with ESRD, previous studies have shown associations between high CTR and malnutrition, inflammation, and fluid overload [18]. In addition, a high CTR has also been strongly associated with a higher incidence and mortality of cardiovascular events [19].

AoAC and CTR have been shown to be positively correlated in patients with CKD [20]. Our recent study also showed that a combination of AoAC score and CTR could predict adverse clinical outcomes, including all-cause mortality, cardiovascular mortality, and a rapid deterioration in renal function in patients with CKD [21]. However, few studies have evaluated the predictive ability of a combination of AoAC score and CTR for clinical outcomes in maintenance hemodialysis (HD) patients. Therefore, the aim of this study was to evaluate the relationships between a combination of AoAC and CTR and overall and cardiovascular mortality in patients with HD. Furthermore, we evaluated the predictive power of AoAC and CTR for clinical outcomes.

## 2. Study Patients and Methods

### 2.1. Study Patients and Design

**Figure 1:**
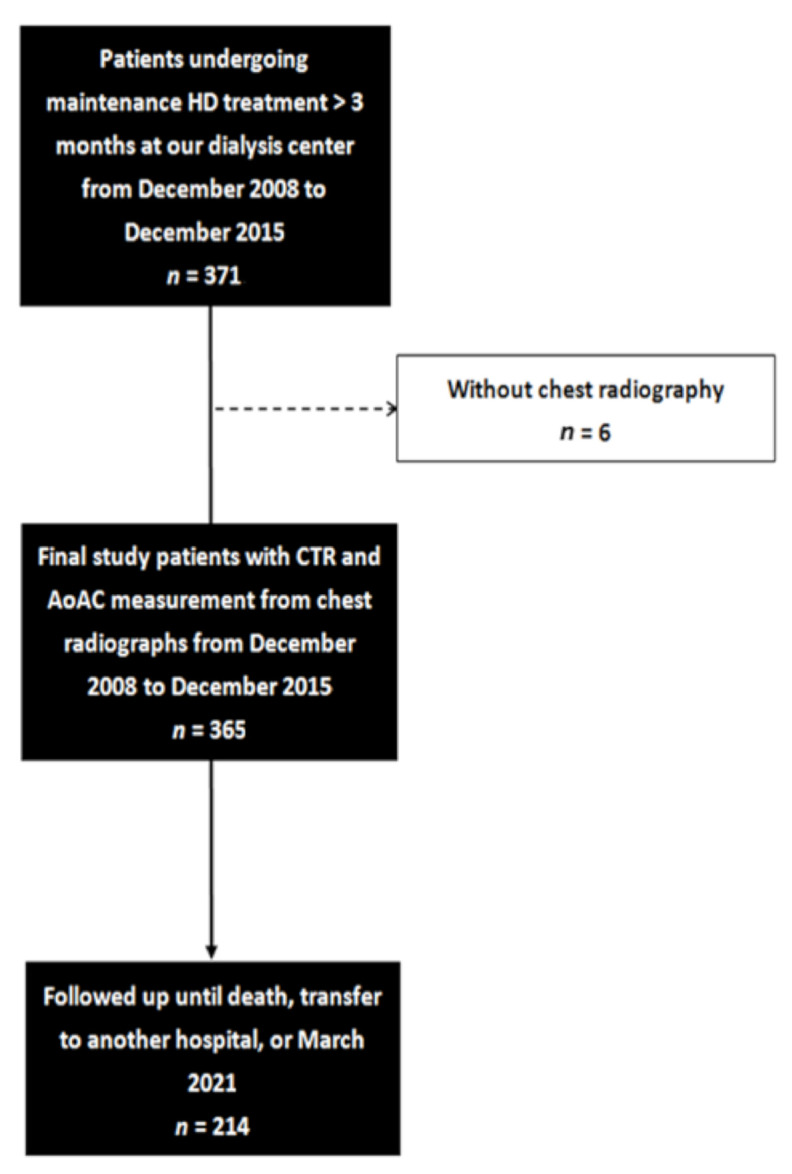
The flow chart of this study.

We conducted a retrospective study about the association between AoAC, CTR on chest X-ray, and mortality in HD patients. A total of 371 patients (191 males and 180 females) were enrolled from a dialysis center of a regional hospital in southern Taiwan from December 2008 to December 2015. All routine HD patients in the hospital except 6 patients without chest radiography were included. Finally, there were 365 patients (188 males and 177 females) included. The patients were followed until death, transfer to another hospital, or March 2021, whichever occurred first (Figure 1). All of the patients had received maintenance HD three times a week for more than 3 months. The Institutional Review Board of Kaohsiung Medical University Hospital approved this study (KMUHIRB-E(II)-20150246 and 22 December 2015 approval), and all participants provided written informed consent. All methods were performed in accordance with relevant guidelines.

### 2.2. Evaluation of AoAC and CTR by Chest X-ray

Chest plain films of the enrolled patients were reviewed by a single experienced radiologist who was blinded to the clinical data of the patients. AoAC was assessed using the scale proposed by Ogawa et al. [8]. The aortic arch as visualized on chest X-ray was divided into 16 sections by circumference, and the number of calcified sections was counted to yield the AoAC score. CTR was calculated as the transverse diameter of the cardiac silhouette divided by that of the chest on a chest X-ray. Cardiomegaly was defined as a CTR > 50%.

### 2.3. Collection of Demographic, Medical and Laboratory Data

Demographic and medical data including age, sex, smoking history (ever vs. never), and comorbidities were obtained from medical records and patient interviews. Fasting blood samples were obtained within 1 month of enrollment into the study, and laboratory data were measured from the samples using an autoanalyzer (COBAS Integra 400, Roche Diagnostics GmbH, D-68298 Mannheim, Germany). Kt/V was calculated using Daugirdas’s formula and used to evaluate the efficiency of dialysis [22]. In addition, information on the medications that the patients were taking during the study period was obtained from their medical records, and included HMG-CoA reductase inhibitors (statins), angiotensin converting enzyme inhibitors (ACEIs), angiotensin II receptor blockers (ARBs), and antiplatelet agents.

### 2.4. Definitions of Overall and Cardiovascular Mortality

The primary and secondary endpoints of the study are overall and cardiovascular mortality, respectively. Two cardiologists identified and confirmed overall and cardiovascular deaths from the patients’ medical records. Disagreements were resolved through consensus with a third cardiologist. These patients received regular HD in our dialysis clinics. During each dialysis, we ask about the patient's condition. If a patient is hospitalized, whether in our hospital or other hospitals, we can obtain detailed information through in-person consultation or telephone consultation.

### 2.5. Reproducibility

Thirty patients were randomly selected, and one experienced radiologist and one trained medical doctor evaluated the reproducibility of their AoAC measurements. The mean percentage error was calculated as the absolute difference divided by the average of two observations.

### 2.6. Statistical Analysis

Statistical analysis was performed using SPSS for Windows version 26.0 (SPSS Inc., Chicago, IL, USA). Data are reported as percentage, mean ± standard deviation, or median (25th–75th percentile) for triglycerides and follow-up period. The patients were stratified into four groups according to a median AoAC score of <3 or ≥3 and CTR < 50% or ≥50%. Multiple comparisons among the study groups were performed using one-way analysis of variance followed by Bonferroni’s post hoc test. The Kaplan–Meier method was used to plot survival curves for overall and cardiovascular survival. Multivariable Cox proportional hazards analysis was used to identify associations between the study groups, AoAC, and CTR with overall and cardiovascular mortality in two models. The first model included the study groups, age, sex, and variables with *p* < 0.05 in univariable analysis. The AoAC < 3 and CTR < 50% group had the lowest risk of mortality and was defined as the reference group. The second model included AoAC, CTR, age, sex and variables with *p* < 0.05 in univariable analysis. The predictive performance of the model for clinical outcomes was assessed using the χ2 test. A difference was considered significant at *p* < 0.05.

## 3. Results

A total of 365 HD patients were included (188 men and 177 women; mean age 58.4 ± 12.3 years). The median AoAC score was 3, and 48.5% of the patients had a CTR ≥ 50%. The mean error of AoAC measurements was 12.3 ± 12.3%. The patients were stratified into four groups according to an AoAC score < 3 or ≥3 and CTR 50% < or ≥50%.

### 3.1. Comparisons of the Clinical Characteristics among the Study Groups

Comparisons of the clinical characteristics among the study groups are shown in Table 1. There were 107, 75, 81 and 102 patients in the four groups, respectively. Compared to the AoAC < 3 and CTR < 50% group, the AoAC ≥ 3 and CTR ≥ 50% group was more predominantly female and had a higher AoAC, higher CTR, older age, higher prevalence of coronary artery disease and cerebrovascular disease and higher prescription rate of antiplatelet agents. Regarding outcomes, compared to the AoAC < 3 and CTR < 50% group, the AoAC ≥ 3 and CTR ≥ 50% group had a shorter follow-up period, and higher overall and cardiovascular mortality.

We estimated that the study has 80% power to detect a relative risk (e.g., ≥2.5) for death using a two independent proportions test with a two-sided significance α level of 0.05.

### 3.2. Risk of Overall Mortality

The median follow-up period was 6.3 (2.7–9.5) years. During the follow-up period, 151 of the 365 patients died (41.4%), including cardiovascular deaths (*n* = 80), malignancy (*n* = 14), infectious diseases (*n* = 38), gastrointestinal bleeding (*n* = 9), and others (*n* = 10).

**Figure 2:**
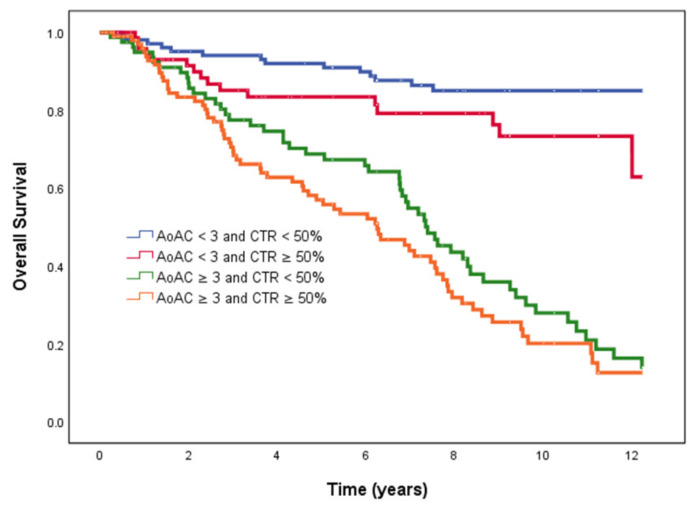
Kaplan–Meier analyses of overall survival among 4 study groups. The group with AoAC < 3 and CTR ≥ 50%, AoAC ≥ 3 and CTR < 50% and AoAC ≥ 3 and CTR ≥ 50% had worse overall survival than that with AoAC < 3 and CTR < 50%.

Figure 2 illustrates the Kaplan–Meier curves of overall survival (log-rank *p* < 0.001) among the four study groups. The AoAC < 3 and CTR ≥ 50%, AoAC ≥ 3 and CTR < 50% and AoAC ≥ 3 and CTR ≥ 50% groups had worse overall survival than the AoAC < 3 and CTR < 50% group.

The results of Cox proportional hazards regression analysis of the four study groups, AoAC and CTR for overall mortality are shown in Table 2. In the univariable analysis, the AoAC < 3 and CTR ≥ 50%, AoAC ≥ 3 and CTR < 50% and AoAC ≥ 3 and CTR ≥ 50% groups (vs. the AoAC < 3 and CTR < 50% group), high AoAC, high CTR old age, smoking history, history of diabetes mellitus, coronary artery disease and cerebrovascular disease, high fasting glucose, and the use of antiplatelet agents were significantly associated with increased overall mortality. In multivariable Model 1, after adjusting for age, sex and the variables with *p* < 0.05 in the univariable analysis, compared to the AoAC < 3 and CTR < 50% group, the AoAC ≥ 3 and CTR < 50% group (hazard ratio [23], 4.576; 95% confidence interval [CI], 2.314 to 9.051; *p* < 0.001), AoAC ≥ 3 and CTR ≥ 50% group (HR, 5.912; 95% CI, 2.968 to 11.776; *p* < 0.001), old age, and diabetes mellitus were significantly associated with increased overall mortality. In Model 2, high AoAC (per 1 score; HR, 1.106; 95% CI, 1.054 to 1.160; *p* < 0.001), high CTR (per 1%; HR, 1.042; 95% CI, 1.010 to 1.076; *p* = 0.011), old age, smoking history, and diabetes mellitus were significantly associated with increased overall mortality.

### 3.3. Incremental Values of CTR and AoAC in Relation to Overall Mortality

**Figure 3:**
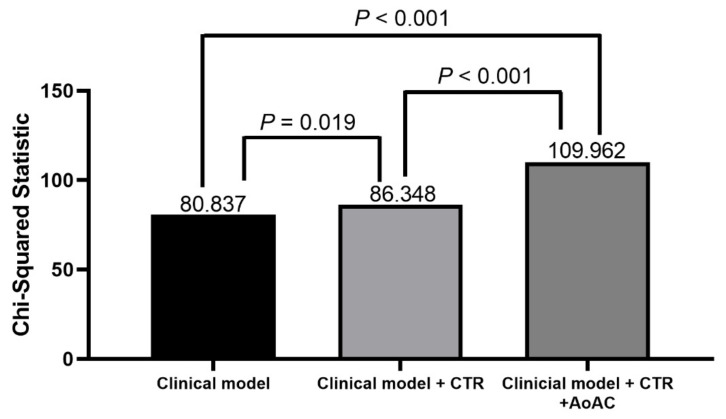
The assessment of predictive model of overall mortality. The clinical model included age, sex, smoking history, diabetes, coronary artery disease, cerebrovascular disease, fasting glucose and antiplatelet agent use (variables in Table 2 of *p* < 0.05 in univariable analysis).

The incremental values of CTR and AoAC in predicting overall mortality are shown in Figure 3. The basic clinical model included age, sex and the variables with *p* < 0.05 in the univariable analysis (Table 2) (χ^2^ = 80.837). Adding CTR to the basic clinical model improved the predictive ability for overall mortality (χ^2^ = 86.348, χ^2^ change = 5.511, *p* = 0.019). In addition, adding AoAC to the previous model (basic clinical model + CTR) resulted in a significant improvement in the prediction of overall mortality (χ^2^ = 109.962, χ^2^ change = 23.614, *p* < 0.001).

### 3.4. Risk of Cardiovascular Mortality

The 80 cardiovascular deaths recorded during follow-up included heart failure (*n* = 35), myocardial infarction (*n* = 12), ventricular fibrillation (*n* = 22) and hemorrhagic stroke (*n* = 11).

**Figure 4:**
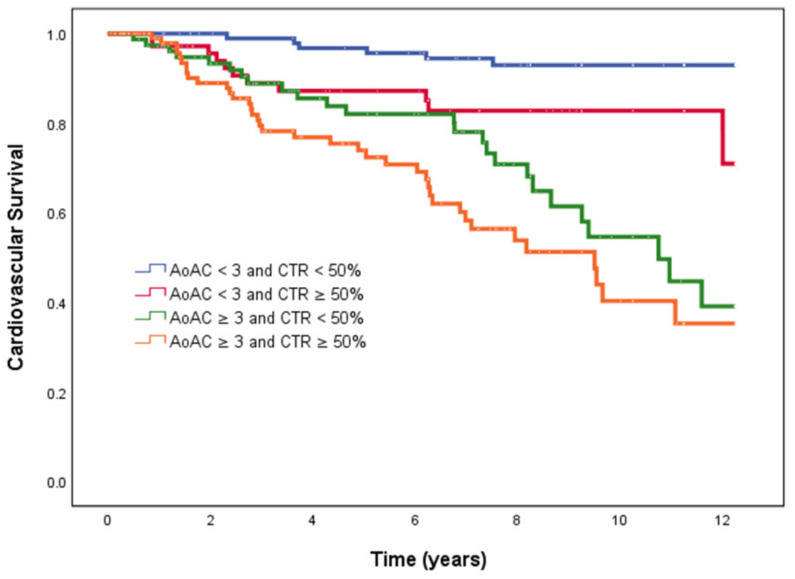
Kaplan–Meier analyses of cardiovascular survival among 4 study groups. The group with AoAC < 3 and CTR ≥ 50%, AoAC ≥ 3 and CTR < 50% and AoAC ≥ 3 and CTR ≥ 50%had worse cardiovascular survival than that with AoAC < 3 and CTR < 50%.

Figure 4 illustrates the Kaplan–Meier curves for overall survival (log-rank *p* < 0.001) among the four study groups. The AoAC < 3 and CTR ≥ 50%, AoAC ≥ 3 and CTR < 50% and AoAC ≥ 3 and CTR ≥ 50% groups had worse overall survival than the AoAC < 3 and CTR < 50% group.

The results of Cox proportional hazards regression analysis of the four study groups, AoAC, and CTR for overall mortality are shown in Table 3. In the univariable analysis, the AoAC < 3 and CTR ≥ 50%, AoAC ≥ 3 and CTR < 50% and AoAC ≥ 3 and CTR ≥ 50% groups (*vs.* the AoAC < 3 and CTR < 50% group), high AoAC, high CTR old age, smoking history, history of diabetes mellitus, coronary artery disease and cerebrovascular disease, high fasting glucose, low total cholesterol, and the use of antiplatelet agents were significantly associated with increased cardiovascular mortality. In multivariable Model 1, after adjusting for age, sex and the variables with *p* < 0.05 in the univariable analysis, compared to the AoAC < 3 and CTR < 50% group, the AoAC < 3 and CTR ≥ 50% group (HR, 3.806; 95% CI, 1.264 to 11.460; *p* = 0.017), AoAC ≥ 3 and CTR < 50% group (HR, 4.993; 95% CI, 1.783 to 13.981; *p* = 0.002), and AoAC ≥ 3 and CTR ≥ 50% group(HR, 8.614; 95% CI, 3.112 to 23.845; *p* < 0.001), smoking history, diabetes mellitus, and coronary artery disease were significantly associated with increased cardiovascular mortality. In Model 2, high AoAC (per 1 score; HR, 1.108; 95% CI, 1.035 to 1.187; *p* = 0.003), high CTR (per 1%; HR, 1.077; 95% CI, 1.039 to 1.116; *p* < 0.001), old age, smoking history, diabetes mellitus, coronary artery disease, and low total cholesterol were significantly associated with increased cardiovascular mortality.

### 3.5. Incremental Values of CTR and AoAC in Relation to Cardiovascular Mortality

**Figure 5:**
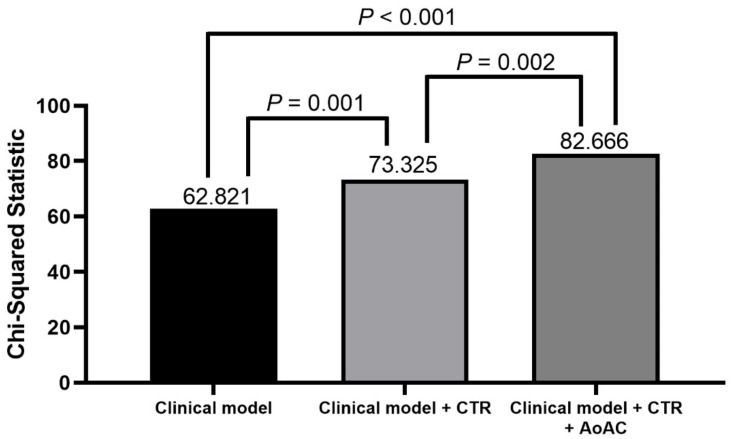
The assessment of predictive model of cardiovascular mortality. The clinical model included age, sex, smoking history, diabetes, coronary artery disease, cerebrovascular disease, fasting glucose, total cholesterol and antiplatelet agent use (variables in Table 3 of *p* < 0.05 in univariable analysis).

The incremental values of CTR and AoAC in predicting cardiovascular mortality are shown in Figure 5. The basic clinical model included age, sex and the variables with *p* < 0.05 in the univariable analysis (Table 3) (χ^2^ = 62.821). Adding CTR to the basic clinical model improved the predictive ability for cardiovascular mortality (χ^2^ = 73.325, χ^2^ change = 10.504, *p* = 0.001). In addition, adding AoAC to the previous model (basic clinical model + CTR) resulted in a significant improvement in the prediction of cardiovascular mortality (χ^2^ = 82.666, χ^2^ change = 9.341, *p* = 0.002).

## 4. Discussion

In this study, we investigated the associations between a combination of AoAC and the presence of cardiomegaly with overall or cardiovascular mortality in 365 patients with HD. We found that the patients with a high AoAC score and cardiomegaly had the highest overall and cardiovascular mortality among the four groups. Furthermore, adding AoAC and CTR improved the predictive ability for overall and cardiovascular mortality in the HD patients.

The first important finding of this study is that a high AoAC score was associated with increased overall and cardiovascular mortality. Moreover, adding AoAC and CTR to the basic clinical model resulted in a significant improvement in the prediction of overall and cardiovascular mortality. VC is a serious and complex issue, and the incidence and severity are much higher in patients with ESRD, especially in those receiving HD, than in the general population [24]. VC can be assessed using radiologic modalities including computed tomography (CT), ultrasound, and plain X-ray [25,26]. Although cardiac CT can be used to quantitatively and accurately assess the extent of cardiovascular calcification, it is costly and may involve the use of contrast media. Plain X-rays are widely available, non-invasive, and relatively inexpensive, and they can be used to quickly assess the degree of calcification of large blood vessels such as the aortic arch, abdominal aorta, iliac artery, femoral artery, radial artery, and even finger arteries [27]. The AoAC score can be calculated from chest X-rays, which represents the extent of VC in the aorta. Many studies have shown that the presence and progression of AoAC are strong risk factors for cardiovascular morbidity and mortality [28,29,30]. The etiology of VC is multifactorial in patients with ESRD, and it has been reported to involve aging, diabetes, smoking, oxidative stress, inflammation, hyperfibrinogenemia, dysregulation of fibroblast growth factor 23, intact parathyroid hormone, calcium and phosphorus, protein-energy wasting, loss of osteopontin and fetuin-A, the osteoprotegerin/receptor activator of NF-kB/receptor activator of the NF-kB ligand system, as well as an increase in sclerostin [31,32,33]. In addition, apoptosis and osteochondrogenic differentiation of vascular smooth muscle cells have also been shown to play important roles in arterial medial calcification, which can induce tunica media remodeling and increase arterial stiffness and reduce vascular compliance [34,35]. An increase in arterial blood pressure and systemic arterial resistance and a reduction in large vessel compliance can result in thickening of myocardial cells and concentric remodeling of the left ventricle. Beyond VC, these patients also have a higher risk of heart valve calcification [36]. Changes in the structure and function of the heart and vessel systems induced by VC can increase the risk of cardiovascular events and death, which may explain the findings in the present study.

The second important finding of this study is that a high CTR was associated with increased overall and cardiovascular mortality. Moreover, adding CTR to the basic clinical model resulted in a significant improvement in the prediction of overall and cardiovascular mortality. Of certain, echocardiography is still a gold standard diagnostic modality for the identification of cardiac structural and functional abnormality. However, in most countries in the world, not all patients undergoing HD can receive echocardiography regularly due to uneven medical resources and health insurance payment issue. Because this examination requires specialized ultrasound machine and well-trained physicians or technicians to perform. CTR derived from chest radiography is a simple way to assess LV size. A CTR greater than 50% is an indicator of cardiomegaly, which is also associated with increased LV mass and LV hypertrophy [37,38]. A higher CTR has also been shown to predict increased mortality in patients with cardiovascular disease [18,19]. In patients with CKD and ESRD, many factors can cause LV hypertrophy, including pressure overload (hypertension, arteriosclerosis, and sometimes aortic stenosis), and volume overload (anemia, arteriovenous fistula, hypervolemia). In persistent LV overload, the hypertrophy can progress to a maladaptive condition, resulting in decreased capillary density in the myocardium as well as increased myocardial cell death and fibrosis, eventually leading to congestive heart failure [39,40]. Therefore, a higher CTR may imply decreased cardiac function and may be associated with a higher risk of cardiovascular mortality. In addition, previous studies have also reported a strong correlation between a high CTR and malnutrition and inflammation in patients undergoing peritoneal dialysis and patients without diabetes undergoing HD [18,41]. Taken together, these findings show that cardiomegaly, as represented by a higher CTR, can be regarded as a meaningful indicator of poor outcomes in patients with ESRD.

Another important finding of this study is that the patients with a combination of a high AoAC score and cardiomegaly had the highest overall and cardiovascular mortality among the four groups. This result suggests that an interaction between VC and LV hypertrophy had an additional effect on the risk of cardiovascular events. Similarly, Hwang et al. reported that VC and LV hypertrophy had a synergistic effect on the risk of death and cardiovascular events in HD patients [42]. Other studies have also speculated that VC and LV hypertrophy may exacerbate each other. VC has been shown to affect the pulsatile dynamics of the vasculature, increase systemic arterial resistance, and consequently contribute to an increase in LV afterload [43]. Increased filling pressure and compensatory myocardial thickening would limit LV diastolic function, which may then lead to LV diastolic stiffness and concentric LV hypertrophy [44]. In contrast, LV hypertrophy may induce greater circulatory stress resulting in impaired endothelial function, which may then cause more severe inflammation and a greater risk of endotoxemia, all of which would contribute to VC [42,45]. One of the major causes of cardiomegaly in ESRD patients is volume overload. Yotsueda et al. proposed that volume overload exacerbates arteriosclerosis through reactive oxygen species-related mechanisms, including inappropriate activation of the renin-angiotensin-aldosterone system and impaired nitric oxide-mediated vascular endothelial dysfunction [19]. This may partly explain the synergetic effect of AoAC and CTR on clinical outcomes.

In addition, the difference in mortality rate between group with AoAC ≥ 3 and CTR < 50% and group with AoAC ≥ 3 and CTR ≥ 50% did not reach statistical significance, which implies that AoAC is a more important factor for overall and cardiovascular mortality than CTR. This may be because cardiomegaly is partially caused by fluid overload. Thus, adequate ultrafiltration can improve hypervolemia status if there are no other contraindications, and then reduce CTR. A previous study also showed that intensive HD can regress LV hypertrophy and decrease mortality [46]. However, whether VC can be reversed is more controversial. Chen et al. suggested that although soluble amorphous calcium-phosphate in uremic-related vascular calcification could be reversed, most of the calcification is extremely insoluble under physiological conditions, and thus it is difficult to completely remove the calcium deposition [47]. Removing the calcium deposition would involve multiple interventions, including surgical or medical parathyroidectomy when indicated, negative calcium and phosphate balance, avoiding calcification inducers, and restoring anti-calcification factor balance. Taken together, the existence of VC may be difficult to change and require a long period of treatment. However, a recent study reported that denosumab treatment could reverse advanced AoAC in HD patients when used for a long time (more than 30 months) [48]. Correcting both VC and cardiomegaly in patients with ESRD can be expected to greatly improve the prognosis.

There are several limitations to this study. First, AoAC and cardiomegaly were determined using plain radiography instead of CT, which is a more accurate method of evaluating the extent of cardiovascular calcification. Plain radiographs may not be sensitive enough to detect the early stage or subtle changes in VC, and the assessment may be affected by body size and overlapping structures. However, plain radiography is a simple and convenient method of assessing cardiomegaly and AoAC, and therefore clinical physicians can easily monitor adverse clinical outcomes related to AoAC and cardiomegaly and follow up. Second, confounding factors including the use of medications with phosphate binders, calcimimetics, 25-hydroxyvitamin D, fetuin-A and osteoprotegerin were not included in our analysis. Third, AoAC and cardiomegaly were measured only once at enrollment. Therefore, we could not evaluate the association between the effects of AoAC and cardiomegaly with the clinical outcomes over time. Finally, the enrollment period is quite long, which would influence the duration of follow-up. Above problem resulted in a non-homogeneous study design.

In conclusion, our results revealed that HD patients with a combination of a high AoAC score and cardiomegaly had the highest overall and cardiovascular mortality. Furthermore, adding AoAC and CTR improved the predictive ability for overall and cardiovascular mortality. Chest radiography can provide a lot of useful prognostic information for patients with HD. Our study raised a solid evidence to remind physicians about the importance of chest radiography evaluation in these patients. In patients with both a high AoAC score and CTR, aggressive management strategies should be arranged to prevent cardiovascular events.

## Figures and Tables

**Table 1:** Comparison of clinical characteristics among study groups.

Characteristics	AoAC < 3 and CTR < 50% (*n* = 107)	AoAC < 3 and CTR ≥ 50% (*n* = 75)	AoAC ≥ 3 and CTR < 50% (*n* = 81)	AoAC ≥ 3 and CTR ≥ 50% (*n* = 102)	*p*
AoAC	0.32 ± 0.71	0.55 ± 0.86	6.69 ± 2.95 *^,†^	7.03 ± 2.94 *^,†^	<0.001
CTR (%)	44.7 ± 3.2	55.1 ± 6.3 *	46.0 ± 2.5 ^†^	55.5 ± 4.4 *^,#^	<0.001
Age (year)	50.1 ± 11.5	5.68 ± 11.2 *	63.2 ± 9.6 *^,†^	64.5 ± 10.4 *^,†^	<0.001
Male gender (%)	65.4	34.7 *	64.2 ^†^	39.2 *^,#^	<0.001
Smoking (ever) (%)	35.8	22.7	37.5	26.7	0.109
Diabetes mellitus (%)	41.1	46.7	54.3	55.9	0.130
Hypertension (%)	78.5	70.7	77.8	82.3	0.325
Coronary artery disease (%)	6.5	16.0	19.8	41.2 *^,†,#^	<0.001
Cerebrovascular disease (%)	0	10.7	14.8 *	11.8 *	0.001
Laboratory parameters					
Fasting glucose (mg/dL)	110.5 ± 44.6	137.7 ± 107.1	132.4 ± 8.02	137.2 ± 69.1	0.033
Triglyceride (mg/dL)	125 (85–187)	131 (89.5–181.5)	132 (84.5–201.5)	155.5 (89.75–197)	0.673
Total cholesterol (mg/dL)	177.6 ± 44.0	186.6 ± 46.2	179.5 ± 62.7	178.4 ± 42.6	0.635
Hemoglobin (g/dL)	10.2 ± 1.1	9.8 ± 1.3	10.3 ± 1.1 ^†^	10.1 ± 1.3	0.025
Total calcium (mg/dL)	9.3 ± 0.8	9.3 ± 0.8	9.5 ± 1.0	9.5 ± 0.9	0.209
Phosphorous (mg/dL)	4.8 ± 1.2	5.0 ± 1.5	4.8 ± 1.3	4.8 ± 1.3	0.644
Calcium-phosphorous product (mg^2^/dL^2^)	44.6 ± 12.3	46.6 ± 14.0	45.6 ± 13.8	45.5 ± 12.0	0.806
Kt/V (Daugirdas)	1.49 ± 0.26	1.60 ± 0.29	1.53 ± 0.25	1.56 ± 0.30	0.084
Medications					
ACEI and/or ARB use (%)	19.4	18.8	16.2	23.9	0.652
Statins use (%)	31.6	23.4	24.3	39.1	0.105
Antiplatelet agent use (%)	4.1	14.1	21.6 *	32.6 *^,†^	<0.001
Outcome					
Follow-up period (years)	8.2 (6.2–12.2)	6.2 (2.3–9.2) *	6.8 (2.6–8.3) *	4.6 (2.4–7.6) *	<0.001
Overall mortality (%)	13.1	21.3	65.4 *^,†^	66.7 *^,†^	<0.001
Cardiovascular mortality (%)	5.6	14.7	30.9 *	37.3 *^,†^	<0.001

Abbreviations. AoAC, aortic arch calcification; CTR, cardiothoracic ratio; ACEI, angiotensin converting enzyme inhibitor; ARB, angiotensin II receptor blocker. Data are reported as percentage, mean ± standard deviation, or median (25th–75th percentile) for triglycerides and follow-up period. The study patients were stratified into 4 groups according to median score of AoAC (3) and CTR ≥ 50% or <50%. * *p* < 0.05 compared AoAC < 3 and CTR < 50%; ^†^ *p* < 0.05 compared with AoAC < 3 and CTR ≥ 50%; ^#^ *p* < 0.05 compared with AoAC ≥ 3 and CTR < 50%. Multiple comparisons among the study groups were performed using one-way analysis of variance followed by Bonferroni’s post hoc test.

**Table 2:** Determinants of overall mortality using Cox proportional hazards model in study patients.

Parameter	Univariable		Multivariable (Model 1)		Multivariable (Model 2)	
	HR (95% CI)	*p*	HR (95% CI)	*p*	HR (95% CI)	*p*
Study group						
AoAC < 3 and CTR < 50%	Reference		Reference		—	—
AoAC < 3 and CTR ≥ 50%	2.249 (1.096–4.615)	0.027	1.739 (0.765–3.950)	0.187	—	—
AoAC ≥ 3 and CTR < 50%	7.482 (4.138–13.529)	<0.001	4.576 (2.314–9.051)	<0.001	—	—
AoAC ≥ 3 and CTR ≥ 50%	9.338 (5.214–16.725)	<0.001	5.912 (2.968–11.776)	<0.001	—	—
AoAC (per 1 score)	1.165 (1.125–1.206)	<0.001	—	—	1.106 (1.054–1.160)	<0.001
CTR (per 1%)	1.049 (1.027–1.071)	<0.001	—	—	1.042 (1.010–1.076)	0.011
Age (per 1 year)	1.062 (1.047–1.078)	<0.001	1.031 (1.012–1.051)	0.002	1.033 (1.013–1.054)	0.001
Gender (male vs. female)	1.157 (0.840–1.592)	0.372	1.021 (0.626–1.666)	0.933	1.148 (0.702–1.876)	0.583
Smoking (ever vs. never)	1.528 (1.089–2.143)	0.014	1.622 (0.957–2.750)	0.072	1.713 (1.016–2.888)	0.043
Diabetes mellitus	2.370 (1.702–3.302)	<0.001	1.566 (1.059–2.315)	0.025	1.660 (1.124–2.451)	0.011
Hypertension	1.095 (0.749–1.599)	0.640	—	—	—	—
Coronary artery disease	1.893 (1.326–2.702)	<0.001	1.364 (0.895–2.077)	0.148	1.484 (0.976–2.257)	0.065
Cerebrovascular disease	2.380 (1.462–3.874)	<0.001	1.352 (0.763–2.399)	0.302	1.352 (0.767–2.383)	0.297
Laboratory parameters						
Fasting glucose (per 1 mg/dL)	1.003 (1.002–1.005)	<0.001	1.002 (0.999–1.004)	0.170	1.002 (1.000–1.004)	0.077
Triglyceride (log per 1 mg/dL)	1.088 (0.610–1.939)	0.775	—	—	—	—
Total cholesterol (per 1 mg/dL)	0.997 (0.993–1.001)	0.997	—	—	—	—
Hemoglobin (per 1 g/dL)	1.040 (0.911–1.188)	0.562	—	—	—	—
Total calcium (per 1 mg/dL)	0.916 (0.757–1.107)	0.363	—	—	—	—
Phosphorous (per 1 mg/dL)	0.977 (0.862–1.108)	0.715	—	—	—	—
Calcium—phosphorous product (per 1 mg^2^/dL^2^)	0.996 (0.983–1.009)	0.511	—	—	—	—
Kt/V (Daugirdas) (per 1)	0.892 (0.489–1.6260	0.709	—	—	—	—
Medications			—	—	—	—
ACEI and/or ARB use	1.238 (0.820–1.870)	0.309	—	—	—	—
Statins use	0.930 (0.640–1.351)	0.702	—	—	—	—
Antiplatelet agent use	2.407 (1.627–3.560)	<0.001	0.924 (0.586–1.457)	0.734	0.956 (0.604–1.512)	0.846

Values expressed as hazard ratio (HR) and 95% confidence interval (CI). Abbreviations. AoAC, aortic arch calcification; CTR, cardiothoracic ratio; ACEI, angiotensin converting enzyme inhibitor; ARB, angiotensin II receptor blocker. Multivariable Cox proportional hazards analysis was used to identify associations between the study groups, AoAC, and CTR with overall mortality. Multivariate model: adjust for age and sex plus variables of *p* < 0.05 in univariable analysis.

**Table 3:** Determinants of cardiovascular mortality using Cox proportional hazards model in study patients.

Parameter	Univariable		Multivariable (Model 1)		Multivariable (Model 2)	
	HR (95% CI)	*p*	HR (95% CI)	*p*	HR (95% CI)	*p*
Study group						
AoAC < 3 and CTR < 50%	Reference		Reference		—	—
AoAC < 3 and CTR ≥ 50%	3.640 (1.343–9.865)	0.011	3.806 (1.264–11.460)	0.017	—	—
AoAC ≥ 3 and CTR < 50%	8.081 (3.302–19.777)	<0.001	4.993 (1.783–13.981)	0.002	—	—
AoAC ≥ 3 and CTR ≥ 50%	11.970 (5.011–28.592)	<0.001	8.614 (3.112–23.845)	<0.001	—	—
AoAC (per 1 score)	1.160 (1.106–1.217)	<0.001	—	—	1.108 (1.035–1.187)	0.003
CTR (per 1%)	1.057 (1.029–1.085)	<0.001	—	—	1.077 (1.039–1.116)	<0.001
Age (per 1 year)	1.061 (1.039–1.082)	<0.001	1.024 (0.997–1.051)	0.086	1.023 (0.995–1.052)	0.111
Gender (male vs. female)	1.191 (0.767–1.848)	0.436	0.728 (0.347–1.526)	0.401	0.861 (0.416–1.781)	0.686
Smoking (ever vs. never)	1.856 (1.178–2.923)	0.008	2.760 (1.289–5.908)	0.009	2.915 (1.380–6.157)	0.005
Diabetes mellitus	3.196 (1.989–5.135)	<0.001	2.013 (1.170–3.464)	0.011	2.041 (1.193–3.493)	0.009
Hypertension	1.123 (0.664–1.900)	0.666	—	—	—	—
Coronary artery disease	2.344 (1.198–4.586)	0.013	1.954 (1.116–3.423)	0.019	2.202 (1.262–3.842)	0.005
Cerebrovascular disease	2.394 (1.502–3.818)	<0.001	1.033 (0.473–2.256)	0.935	1.084 (0.497–2.363)	0.839
Laboratory parameters						
Fasting glucose (per 1 mg/dL)	1.004 (1.001–1.006)	0.001	1.002 (0.999–1.005)	0.270	1.003 (1.000–1.006)	0.075
Triglyceride (log per 1 mg/dL)	0.934 (0.420–2.076)	0.866	—	—	—	—
Total cholesterol (per 1 mg/dL)	0.994 (0.989–1.000)	0.038	0.995 (0.989–1.001)	0.124	0.993 (0.986–1.000)	0.044
Hemoglobin (per 1 g/dL)	1.138 (0.950–1.363)	0.161	—	—	—	—
Total calcium (per 1 mg/dL)	0.925 (0.712–1.200)	0.925	—	—	—	—
Phosphorous (per 1 mg/dL)	0.943 (0.791–1.125)	0.517	—	—	—	—
Calcium-phosphorous product (per 1 mg^2^/dL^2^)	0.991 (0.973–1.009)	0.341	—	—	—	—
Kt/V (Daugirdas) (per 1)	0.482 (0.204–1.135)	0.095	—	—	—	—
Medications			—	—	—	—
ACEI and/or ARB use	1.127 (0.639–1.988)	0.679	—	—	—	—
Statins use	0.884 (0.533–1.466)	0.633	—	—	—	—
Antiplatelet agent use	3.046 (1.848–5.020)	< 0.001	0.994 (0.555–1.781)	0.984	0.954 (0.529–1.722)	0.877

Values expressed as hazard ratio (HR) and 95% confidence interval (CI). Abbreviations. AoAC, aortic arch calcification; CTR, cardiothoracic ratio; ACEI, angiotensin converting enzyme inhibitor; ARB, angiotensin II receptor blocker. Multivariable Cox proportional hazards analysis was used to identify associations between the study groups, AoAC, and CTR with cardiovascular mortality. Multivariate model: adjust for age and sex plus variables of *p* < 0.05 in univariable analysis.

## Data Availability

Data may be available upon request to interested researchers. Please send data requests to: Szu-Chia Chen. Division of Nephrology, Department of Internal Medicine, Kaohsiung Medical University Hospital, Kaohsiung Medical University.
